# Costing Alternative Birth Settings for Women at Low Risk of Complications: A Systematic Review

**DOI:** 10.1371/journal.pone.0149463

**Published:** 2016-02-18

**Authors:** Vanessa Scarf, Christine Catling, Rosalie Viney, Caroline Homer

**Affiliations:** 1 Centre for Midwifery, Child and Family Heath, University of Technology Sydney, New South Wales, Australia; 2 Centre for Health Economic Research and Evaluation, University of Technology Sydney, New South Wales, Australia; National Institute of Health, ITALY

## Abstract

**Background:**

There is demand from women for alternatives to giving birth in a standard hospital setting however access to these services is limited. This systematic review examines the literature relating to the economic evaluations of birth setting for women at low risk of complications.

**Methods:**

Searches of the literature to identify economic evaluations of different birth settings of the following electronic databases: MEDLINE, CINAHL, EconLit, Business Source Complete and Maternity and Infant care. Relevant English language publications were chosen using keywords and MeSH terms between 1995 and 2015. Inclusion criteria included studies focussing on the comparison of birth setting. Data were extracted with respect to study design, perspective, PICO principles, and resource use and cost data.

**Results:**

Eleven studies were included from Australia, Canada, the Netherlands, Norway, the USA, and the UK. Four studies compared costs between homebirth and the hospital setting and the remaining seven focussed on the cost of birth centre care and the hospital setting. Six studies used a cost-effectiveness analysis and the remaining five studies used cost analysis and cost comparison methods. Eight of the 11 studies found a cost saving in the alternative settings. Two found no difference in the cost of the alternative settings and one found an increase in birth centre care.

**Conclusions:**

There are few studies that compare the cost of birth setting. The variation in the results may be attributable to the cost data collection processes, difference in health systems and differences in which costs were included. A better understanding of the cost of birth setting is needed to inform policy makers and service providers.

## Background

Maternity services are the third most common specialist service in Australia and single spontaneous delivery is the most common principle diagnosis among acute overnight admissions to hospital.[1] There are over 300,000 births in Australia each year [2], with over 99% occurring in a public or private hospital setting, or in a birth centre, leaving less than 1% of women giving birth at home.

In 2009, the Department for Health and Ageing released the National Review of Maternity Services which contained feedback from stakeholders outlining issues relating to limited access to different models of care. [3] As a result, the National Maternity Services Plan [4] was released in 2011 with priorities for the following five years. One of these was to “increase access for Australian women and their family members to local maternity care by expanding the range of models of care” going on to state that “continuing to provide a range of maternity care options, including homebirth, is a priority” (page 31).

The majority of Australian women do not have access to alternative birth settings. Access to birth centres remains at around 5% of women and homebirth less than 1%.[5] Publicly-funded homebirth models have been established around the country but still cater for very small numbers of women, in fact less than 2000 women over a six year period. [6] In a recent analysis of population data in NSW [7] from 2000 to 2008, the vast majority of healthy low risk women (around 94%) gave birth in a hospital labour ward. Other places of birth were home (0.3%), attended by a public or private midwife; or a birth centre (5.6%), most often co-located on the campus of a public maternity service and staffed by midwives.

There is evidence that alternative models of care and settings for birth are a safe, highly acceptable option for childbearing women. Within the last decade, Australian research has shown significantly lower perinatal mortality for women cared for in birth centres compared to hospital births. [8] Significantly higher spontaneous birth rates, lower caesarean section rates and admissions to special care nurseries have also been found in a study of two freestanding birth centres in NSW.[9] In a review of 12 publicly-funded homebirth models from 2005 to 2009 [6] a normal birth rate of 90% was reported with no significant increase in perinatal mortality associated with planned homebirth.

Access to alternative birth settings internationally varies both within and between countries and this is closely linked to status and role of the midwife in that country [10, 11] which is influenced by cultural values, social norms, legislation, education, and the consumer interest. [11] Of the countries included in this review, maternity care in the United Kingdom is the most similar to Australia. The National Health Service (NHS) provides maternity care which is free at point of care to the vast majority of childbearing women. In 2007, the proportion of women giving birth across the settings is as follows: 8% gave birth outside a hospital based maternity unit (obstetric unit), 2.8% at home, and the remainder in a birth centre, either alongside (AMU) an obstetric unit or freestanding (FMU). [12] The Netherlands has an extensive primary health care service which provides out-of-hospital birth services (at home or in a short-stay hospital setting) to women at low risk of complications and has a homebirth rate of approximately 29%. [13] These low-risk women are under the care of a midwife or general practitioner who refer any women with medical complications to specialist obstetric care in a secondary care setting (ie hospital). [14]

In Norway, midwives provide care to all childbearing women in labour in and out of hospital with referral to medical specialists for complicated cases. In-hospital birth accounts for 98.4% of women, the remaining 1.6% of women giving birth out of hospital including at home or in “freestanding birth homes”. [15] The proportion of women giving birth at home in Ireland is 0.2% and these births are attended by self-employed midwives [16]. Midwifery-led units, an out-of hospital birth option, have more recently been implemented however the number of births in these facilities is reported as part of the hospital birth statistics [16] therefore it is not possible to report these statistics.

The total number of out-of-hospital births in the Unites States (US) in 2012 was 1.36%, two-thirds of which occurred at home and the remaining third in birth centres. [17] While this is a national statistic, the state by state proportions vary from 2% to 6% largely due to availability of midwives (state laws vary regarding midwifery and credentialing) and birth centres. The number of birth centres increased to 310 in 2015 [18] however 13 states do not offer birth centre facilities at all. [17] Out-of-hospital birth options in Canada also vary by province. Midwifery regulation was first introduced in 1994 in Ontario and now out of hospital birth is offered six of the 13 provinces. For example, in British Columbia, 11% of births are attended by a midwife and 20% of these are at home [19], and in Ontario, home births comprise 1.6% of the births in the province. [20]

Whilst evidence of the safety of alternative birth settings in Australia and internationally is growing, information about the comparative costs and cost-effectiveness is less easy to find. The most comprehensive study to date has been the Birthplace in England Study [21], in which a birth place cost-effectiveness analysis was undertaken. This prospective cohort study examined the outcomes of 64,538 women and babies according to intended place of birth at the onset of labour and found that it was less expensive for the health system for low risk women to give birth at home, especially for women having their second or subsequent baby. [21] The cost analyses performed in Australia to date have examined the model of care [22–24] or public versus privately provided care [24, 25], but they do not directly address the comparative costs for women of similar risk in different settings. In particular, these studies do not address the question of the comparative cost to the health system in Australia of giving birth at home or in a birth centre compared to a standard hospital labour ward for women at low risk of complications.

The aims of this review are firstly to identify economic evaluations or cost analyses comparing places of birth which include home, birth centres, both freestanding and alongside, and standard hospital labour wards. Secondly, this review aimed to explore the methodological approaches employed by the selected studies. The purpose of this aim was to assess the factors that inform the cost and cost effectiveness of these settings and to determine the most appropriate approach to inform future cost analyses on place of birth.

## Methods

### Search Method

**Fig 1:**
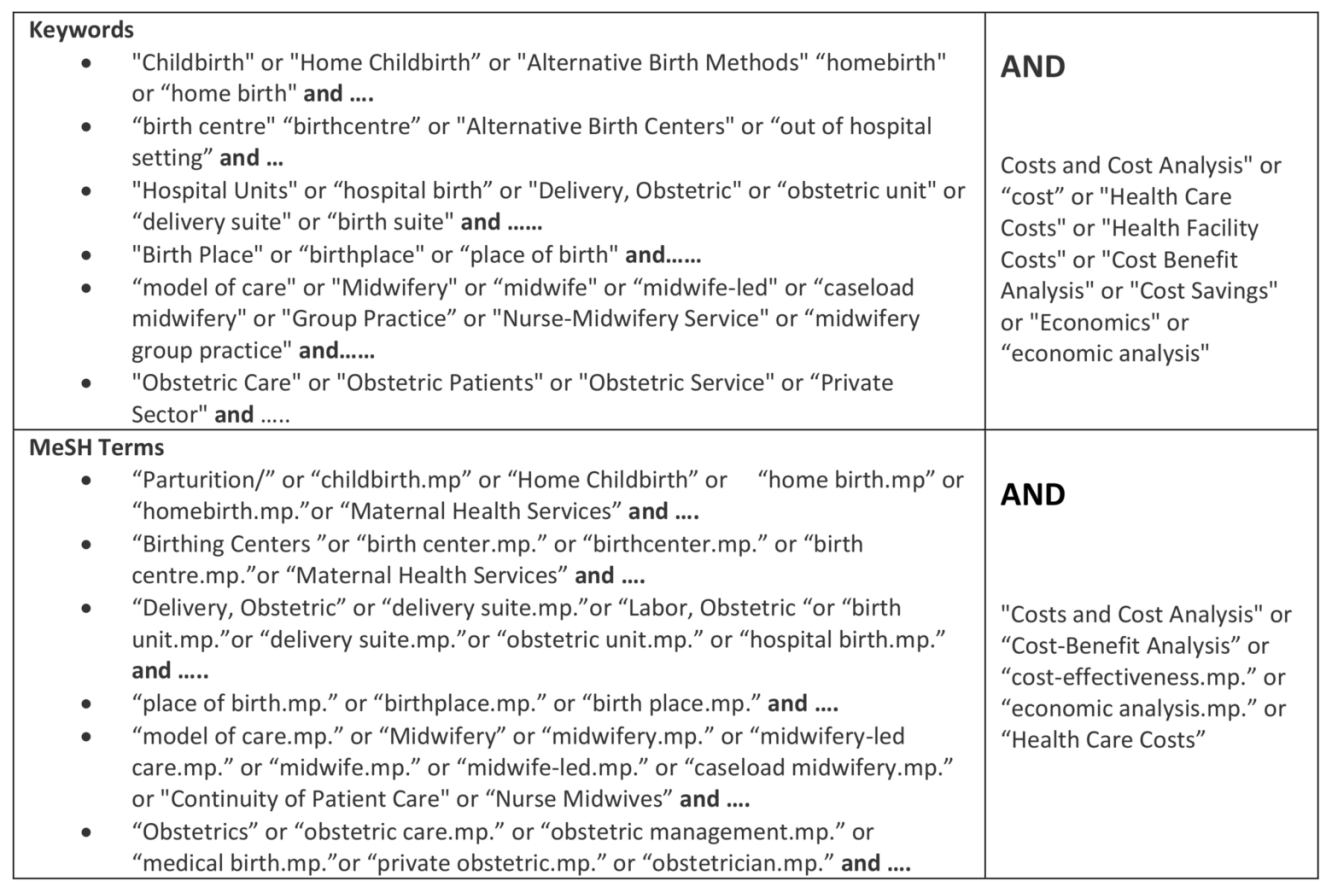
Keywords and MeSH terms used in search strategy.

Searches of the literature to identify economic evaluations of different birth settings were made in February 2015 of the following electronic databases: MEDLINE, CINAHL, EconLit, Business Source Complete and Maternity and Infant care. The PICO principles (population, intervention, comparison, and outcome) were used to inform keyword search terms. [26, 27] Reference lists of relevant articles, including reviews were also examined. The keywords and MeSH subject headings that were used are shown in Fig 1. Due to the multifactorial nature of the subject, search terms were divided into groups, namely model of care and place of birth. These groups were then combined with search terms describing cost analysis or economic analysis separately.

### Search Protocol

**Fig 2:**
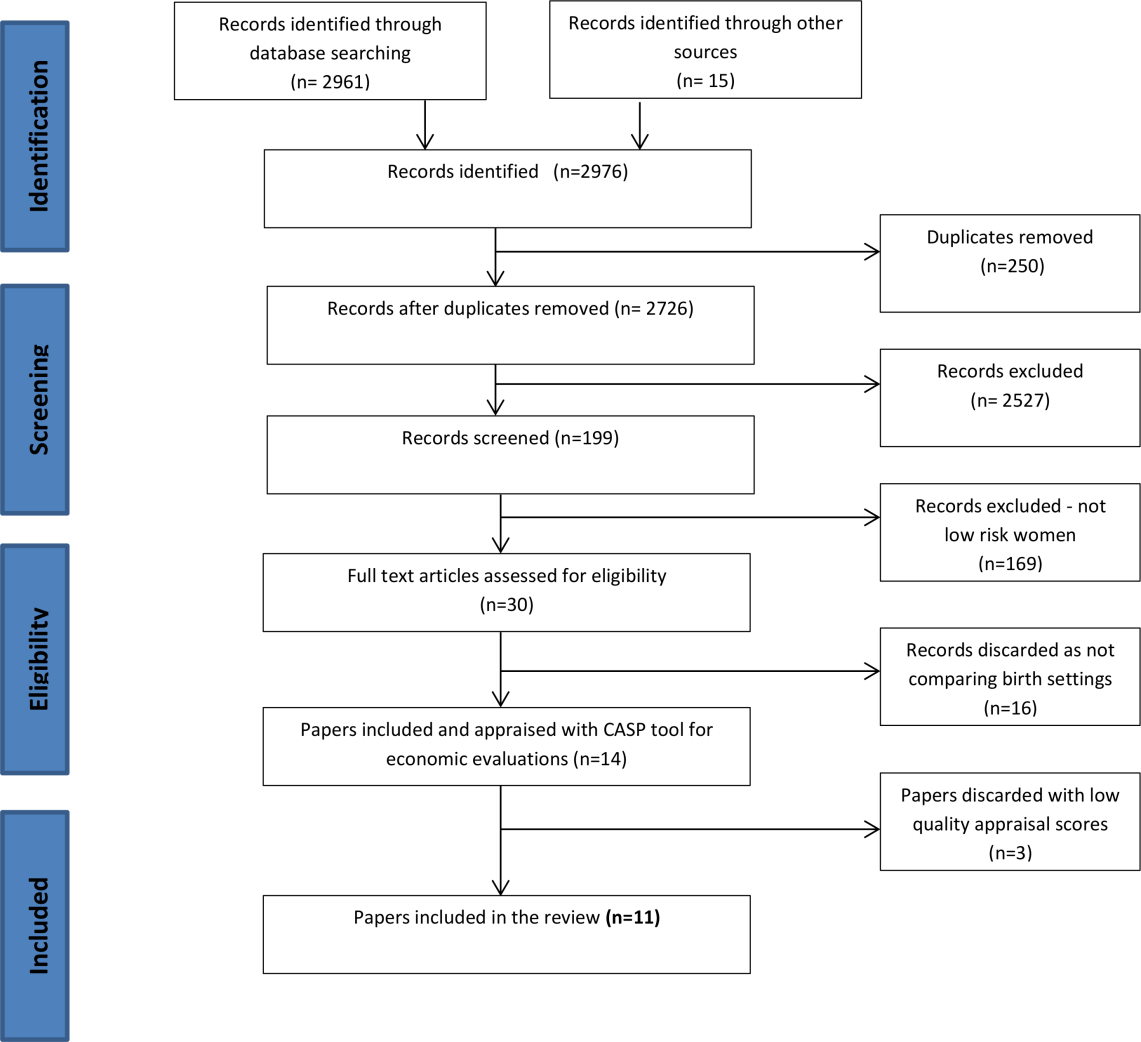
Flowchart of literature review process.

Original, English language studies between the years 1995 and 2015 were included. Studies that reported on costs and/or incorporated an economic analysis of models of care and place of birth for low risk women at term were reviewed. To achieve this, the search included alternative birth settings, models of care, and economic analyses. After testing a number of combinations, the search terms associated with the concepts of place of birth and model of care were paired by group with economic analyses to achieve a reasonable number of papers to review (see Fig 1). Retrieved articles were screened by two authors for their focus on the cost of place of birth specifically comparing two or more birth settings. The other critical factor in the selection of the studies was the focus on a population of women at low risk of complications. This resulted in the close appraisal of 14 articles using the Critical Appraisal Skills Program (CASP) tool for Economic Analyses. Three studies were excluded due to low appraisal scores. Fig 2 is a flowchart of search process.

### Quality Appraisal

**Fig 3:**
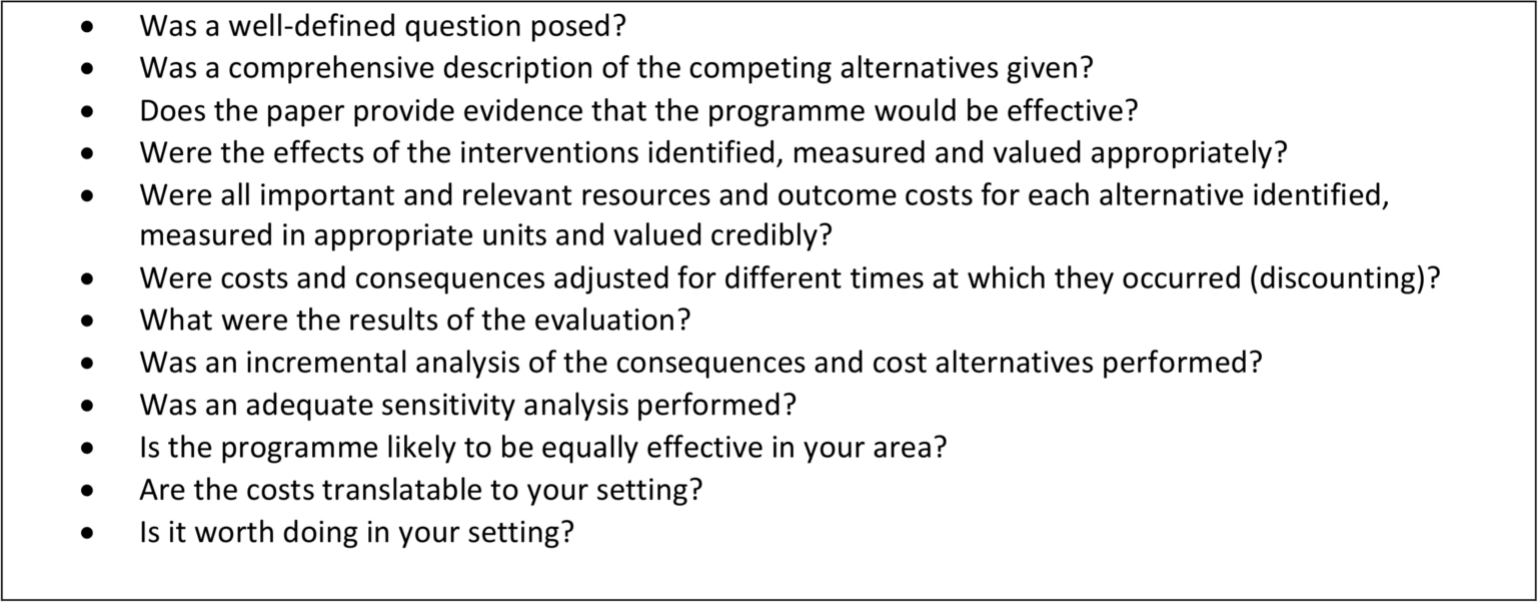
CASP questions.

The Critical Appraisal Skills Programme (CASP) Economic Evaluations Checklist was used to appraise the methodologies of the included papers. This checklist has twelve questions adapted from Drummond et al (2005). The tool consists of questions with some guidance comments where applicable and “Yes, Can’t tell, No” responses (see Fig 3). The question “What were the results of the evaluation?” did not fit the yes/no answer format therefore the overall scores added to eight.

Data were extracted by one reviewer using a template agreed on by all authors that included the following areas of interest: Study design and perspective, population, intervention, comparison and outcome (PICO), source of resource use data, details of cost included in the study, results of the cost analyses, and overall interpretation the results (Table 1).

**Table 1:** Details of included studies in alphabetical order.

First Author Date(country)	Study designPerspective	Population(N =)	Intervention	Comparator	Outcome	Source of resource use data	Included costs	Cost analysis/ cost-effectiveness results	Interpretation
1. Anderson 1999(USA)	Cost-effectiveness analysis- Cohort studyPerspective: Medicaid/Government	Low risk women, (undefined criteria) in 1996 (N = 23 380)	Intended homebirths between 1987 and 1991 attended by CNM	Hospital births unspecified attendant	Birth without intrapartum fetal or neonatal mortality	Retrospective questionnaire to Certified Nurse Midwives,Health Insurance Assoc. of America (HIAA) and other literature for medical charges	CNMs—average charges for performing Home Birth (unspecified)Medical costs estimated from average of published rates charged and the Health Insurance Association of America	Cost comparison- HB $1711 (1991)Hospital $5382 (1991)	HB is a cost effective alternative (estimated saving of 76% relative to hospital birth 1998) with lower rates of neonatal mortality and caesarean section.
2. Bernitz 2012 (Norway)	Cost-effectiveness analysis of RCT Perspective: Health care provider	Low risk women, (MU selection criteria used) between 2006 and 2010 (N = 1110)	Alongside Midwifery Unit (MU) (Birth centre) care by midwives between 2006 and 2010	Standard obstetric unit within same hospital care by midwives	Clinical procedures avoided- LSCS, Instrumental vaginal deliveries, interventions requiring operating room, EDB, augmentation of labour	Hospital accounting, activity databases and patient records	Length of stay, patient activities, service costs and patient related costs, staff resources (average), intervention costs	Cost per patient calculated (4 steps).Top-down/bottom-up. €1672 v €1950ICER- MU dominant strategy	MU less expensive and fewer epidural blocks and augmentation of labour. Measured costs related only to birth care.
3. Hendrix 2009(Netherlands)	Cost analysis of a Prospective non-randomised controlled studyPerspective: Societal costs	low risk women (nulliparous, no indication for secondary care) in 2007(N = 418)	Intended birth at home with a midwife in 2007	Intended birth in a short stay (SCU) ‘hospital setting’ with a midwife or birth in a hospital (OU)	Outcomes not reported	Cost diaries, questionnaires and birth registration forms	Means reported on professional services, procedures (USS), therapy (physio, lactation) delivery mode, Length of stay.	Sensitivity and bootstrappingHome: €3173SCU: €2816 OU: €5208	No difference in cost or consequences between home and the short stay unit.
4.Howell 2014(USA)	Cost analysis–propensity score reweighting model Perspective: Medicaid	Low risk women, (statistically matched for observable characteristics) between 2005 and 2008(N = 43859)	Planned birth in the midwife-led “Family Health and Birth Centre” (FHBC) in 2008	Planned birth in local district hospitals	Reported in another paper: FHBC care less likely to have an LSCS, more likely to have term baby and give birth over the weekend (suggestive of less intervention overall)	Cost estimates from National average Medicaid physician fees, centres for Medicare and Medicaid services (MW), DRG- average cost per hospital stay for delivery mode,NICU costs.	Antenatal, delivery and postnatal care (physician and midwives) average cost per hospital stay (DRGs), normal vaginal birth, caesarean section, admission to NICU	BC care $6055 v Hospital care $7218 (difference $1163 / delivery).	16% reduction in costs for every pregnancy followed in a BC ($11.64 Mill / 10000 Medicaid births). This model could have a significant impact on the cost of the Medicaid obstetric episode
Hundley 1995(Scotland)	Cost analysis of RCT Perspective: Health System/ hospital	Low risk women (criteria not stated) between 1990 and 1992(N = 2844)	Labour and delivery care at midwife-led unit (MU)(costs valued at 1992–1993 UK costs)	Labour and delivery care in a consultant-led unit	Reported in another paper: No significant difference in mode of birth and fetal outcome. High transfer rate for nulliparous women.	Questionnaire by midwife, client (demographics). Medical record review.	Interventions, labour care, pay grade and time spent, consumables, pain relief, (market values). Staff costs and capital costs.	Cost comparison9 scenarios in sensitivity analysis giving different results.	Net increase in cost per women of £40.71 attributable to staff cost. Reduction in cost of consumables in MU.
6. Janssen 2015(Canada)	Cost analysisPerspective: Government Payer	Low risk women (eligibility requirements for a homebirth) between 2001 and 2004 (N = 9864)	Planned homebirth, care provided by a registered midwife	Planned hospital birth care provided by a midwife or a physician	No clinical comparisons made	Linked data from administrative data sources	Fee payments to physicians and midwives from Medical Services plan (MSP), emergency transport costs, hospitalisation cost, and pharmaceuticals.	Average costsPlanned HB: $2275Hospital MW: $4613Hospital Physician: $4816	HB less expensive followed by MW care in hospital
Kenny 2015(Ireland)	Cost Comparison alongside pragmatic Randomised trialPerspective: Health Service	Low risk women (criteria not stated) between 2004 and 2007 (N = 1653)	Midwife-led care in one of two alongside midwifery units (BC) between 2004 and 2007	Consultant-led care in hospital setting	Reported in another paper: no statistically significant difference in outcomes between MLU and CLU for LSCS, induction of labour episiotomy, instrumental birth, low APGARs, and postpartum haemorrhage. Significantly less likely to have electronic fetal monitoring or augmentation of labour.	Facility-based financial information. Verified with financial and services managers.	Medical and midwifery staff costs including antenatal, intrapartum and postnatal care, investigations, interventions, inpatient stay, administrative costs (managers), overheads in the form of general administration and maintenance based on floor space occupied	Average cost: MLU: €2598 CLU: €2780	MLU less expensive
8. Reinharz 2000 (Canada)	Cost-effectiveness analysis—observational cohort studyPerspective: Social	Low risk women (criteria not stated) between 1995 and 1996(N = 2000)	Midwife-led care in 7 birth centres (BC) (pilot project) between 1995–1996	Matched with women who gave birth in hospital under physician care	BC care associated with higher satisfaction, fewer interventions, fewer LSCS, less severe perineal trauma, fewer low birth weight and pre-term infants but trend towards higher stillbirth rate and neonatal resuscitation	Hospital files, physician billing data (Regie de l’assurance maladie du Quebec RAMQ)	Staff salaries, fees for services, minimum wage for women, average staff salaries, fees for services, time spent by women receiving BC services, pharmaceuticals, non-physician services received (eg.chiropractor, dietician), cost to women and significant others of time spent away from regular activities	Cost effectivenessDirect costsBC: Can$2294 ($2062–2930) LW: Can$3020 ($3016- $3017)	Results differed across pilot projects. Intervention not standardised, 7 centres included. Physician costs difficult to access, possible selection bias.
9. Schroeder 2012 (UK)	Cost-effectiveness analysis -Prospective Cohort StudyPerspective: Health system (NHS)	Low risk women (NICE guidelines for definition) between 2008 and 2010(N = 64 538)	Planned birth at home, in an alongside midwifery nit (AMU) or freestanding midwifery unit (FMU) with midwifery care	Planned birth in an obstetric unit	Primary: adverse perinatal outcomes avoided. Secondary: maternal morbidity and number of normal births. No significant differences in the odds of adverse perinatal outcomes for planned births in any of the non-obstetric unit settings compared with the obstetric units.	Data collection forms, supplemental forms and expert-opinion focus groups.	Staffing costs, treatment, surgeries, investigations, medications.	Mean costs Home: £1067AMU: £1435 FMU: £1461Hospital: £1631ICER	Overall, planned HB generated greatest mean net benefit.
Stone 1995(USA)	Cost-effectiveness analysis- decision analytic modelPerspective: Insurer	Low risk women (various definitions) who gave birth in 1986(N = 14070)	Planned birth at a free-standing BC under the care of a midwife	Planned birth at a standard hospital facility under the care of a MW and physician	Data obtained from National Birth centre Study, crude measures in units of utility.	Field interviews of financial managers, ambulance officers, DRGs	Direct costs eg, interventions, provider fees.Indirect costs eg. Fixed equipment costs, education of clinician, patient charges, ambulance transfer charges, published averages.	BC:$3385Hospital:$4673	BC remains cost effective until transfer rate exceeds 62%
Toohill 2012(Australia)	Cost effectiveness-Cohort studyPerspective: Health care system	Low risk women (met birth centre eligibility criteria at one hospital) in 2008(N = 102)	Planned birth in a midwife-led birth centre (BC)	Planned birth in a standard hospital unit	Women in the BC were less likely to have their labour induced, use pharmacological pain relief and caesarean section and more likely to breastfeed. Babies born in the hospital unit were four times more likely to be admitted to the special care nursery.	AR-DRGs, hospital costs attributed to the admission (mother and baby), personal diaries to record visits, Medicare Benefits Schedule for the GP visits.	Care provider time (midwifery and medical) costs, hospital costs, all costs attached to the hospital from 36/40 to 6/52 PN.	MGP:$4722, Standard Care: $5641	MGP care and BC delivery less costly with better outcomes

## Results

Table 1 summarises the included studies. The studies in this analysis had a high degree of heterogeneity in their methods, included costs and aims. The time horizon for all 14 studies was less than one year, therefore discounting was not necessary. All papers scored a ‘no’ for this question on the CASP evaluation tool.

Of the 11 articles reviewed, three were from the United States of America (USA), two from Canada, two from the United Kingdom (UK), two from Europe, one from Ireland and one from Australia. Six of the studies used a cost-effectiveness model, with the remaining five studies reporting a combination of cost analyses (four) and a cost comparison. Eight of the 11 studies found a cost saving in the alternative settings, namely at home or in midwifery-led units (also referred to as birth centres). Two found no difference in the cost of the alternative settings and one found an increase in cost in providing care to women in a birth centre. The intervention compared in this review was the alternative setting for birth and this included home (four studies), birth centres or midwifery-led units (eight studies, Schroeder et al compared both home and birth centres) which were situated either alongside a standard hospital facility (six) or as free-standing facilities separate from a hospital campus (three).

While papers were selected on the basis of including women at low risk of complications, the assessment of low risk status varied among the studies. Women were largely chosen using the criteria for selection to ‘birth centre care’ or ‘midwife-led care’ or ‘eligibility for a homebirth’ and this criteria was used to match the comparison cohort attending the standard hospital labour ward or ‘consultant led unit’. These criteria were not routinely described in the papers therefore it is difficult to attest to the comparability of the cohorts. Schroeder et al (2012) employed the National Institute for Health and Care Excellence (NICE) definition of a woman at low risk of complications.

The perspectives of the studies included societal [14, 28], Medicaid or the Government [19, 21, 23, 29, 30], the individual health service [31, 32] and the insurer [33]. The variation in the perspective was reflected in the identification and collection of the resource use data to measure the costs of the alternative birth settings. The majority of studies found a cost saving in providing pregnancy and birth care to women however the variation in the parameters measured leave questions as to the comparability and generalisability of these economic evaluations.

Many of the studies included in this review have reported clinical outcomes, either from Randomised Controlled Trials (RCTs) or associated studies, however this review has focussed on the cost outcomes of each study. These will be further divided by comparisons of birthplace and cost analysis methodology.

### Comparisons of birthplace

#### Homebirth vs birth centre vs standard labour ward

Four studies described cost analysis or economic analysis of homebirth, birth centres and labour wards for low-risk women. [14, 19, 21, 29] These varied considerably in terms of the sample size and methods. The three larger studies concluded that home birth was less costly and the fourth, which was much smaller, found no difference in cost. Schroeder et al (2012) compared birth at home, alongside midwifery units (AMU), free-standing midwifery units (FSU) and standard obstetric units (OU). They found that homebirth conferred the greatest benefit overall, compared with birth centres and labour wards. This involved a saving of £565 when women gave birth at home compared to the hospital obstetric unit and savings of £195 and £170 when compared to freestanding birth centres and alongside birth centres respectively.

In another large study of 11,592 women who gave birth at home and 11,788 in hospital In the United States, Anderson and Anderson (1999) compared mortality and caesarean section rates and costs of homebirth and hospital birth. The authors concluded that homebirth was less expensive at US$1711 compared to birth in hospital estimated at US$5382. Janssen et al (2015) in British Columbia linked data from six administrative databases from 2001 to 2004 of 9864 women who planned to give birth at home under the care of a “regulated” midwife, or in a hospital under the care of a midwife or a physician. All women met the criteria for home birth eligibility. This study demonstrated significant cost saving between birth settings, home birth being the least costly at CAN$2275, followed by planned hospital birth with a midwife CAN$4613 and hospital birth with a physician CAN$4816 [19]. The smallest study, a prospective non-randomised cost analysis of 418 women conducted in the Netherlands, found no significant difference in the cost between the alternative birth settings. [14]

#### Birth centres vs standard labour wards

The remaining studies examined cost in birth centres and standard labour wards. [23, 28, 30, 33] The majority of these found a cost saving in providing birth centre care [23, 30–33], one study reported no difference in cost [28] and a study in Scotland found a net increase in cost per woman however, this study included the costs of introducing this new model of care and birth setting. [34] Again, the sample sizes and methods varied considerably. Differences included the type of alternative facility, namely alongside or free-standing birth centres, and the recruitment of the women was largely based on vaguely reported criteria.

Toohill (2012) in a small Australian cohort study of 102 women compared the costs and outcomes of women receiving Midwifery Group Practice (MGP) care, who gave birth in a birth centre, with women receiving ‘standard care’ by midwives or GPs, who gave birth in a standard labour ward. When the costs were compared overall, there was a significant reduction in the cost of giving birth in the birth centre under MGP care compared to standard care at AU$4722 and AU$5614 respectively (p<0.001). Further differences in costs between the settings were found in studies by Bernitz (2012) in Norway, and Howell (2014) in the USA. Bernitz studied 1100 women in a 4-year RCT, calculated costs per woman (using a top-down and bottom-up approach), and found women who had care in an alongside midwifery unit (AMU) were less costly to care for than those in the standard obstetric unit (SCU) (€1672 vs €1950 p<0.001) in the same hospital complex. An RCT in Ireland also found the cost of care of women in alongside Midwifery-Led Units (MLU) was €182 lower than in the hospital setting led by consultant doctors. [32]

The cost-analysis by Howell (2014) involved 43,859 participants, 872 of whom gave birth in one birth centre and 42,987 who gave birth in a ‘usual care’ setting who were matched on observable variables on birth certificate data by propensity score modelling. This study found a 16% reduction in costs for every pregnancy conducted within a birth centre; a saving of US$1163 per birth. [30]

In Canada, Reinharz (2000) in a control-matched cohort study measured the cost effectiveness of 2000 women in seven pilot projects and found differing results across all settings. These projects examined the processes and outcomes of women who chose to give birth in midwife-led birth centres compared to women giving birth under the care of midwives or physicians in a hospital setting. The findings varied in cost and effectiveness, with overall conclusions suggesting that midwives are less costly in three of the seven birth centres and in the remaining four, cost overlaps suggested no real cost difference. Sensitivity analyses revealed that the upper limit of the midwifery groups’ costs resulted in a marginally lower cost than the physician costs. [28]

The Scottish RCT by Hundley et al (1995) with 2844 participants examined intrapartum costs of introducing a midwifery-led birth centre compared to the standard consultant-led labour ward. Nine sensitivity analyses of differing factors were tested and the result indicated an additional cost of 10.5% for the introduction of the midwifery-led service (with a range of a 2.5% saving to 11% additional costs). The birth centre was newly established and the results of the trial showed that the setup costs of the birth centre and the higher level of staff employed to staff the new unit factored significantly in the overall cost thus making it a slightly more costly option. This was the only study that showed the costs associated with an alternative birth setting were higher than the standard hospital setting.

Using a decision analytic model, Stone and Walker (1995) measured the cost-effectiveness of birth centre birth compared to hospital birth for women with matched low risk profiles (11,814 birth centre births and 2256 hospital births). The analysis concluded that it was less costly to give birth in a birth centre (US$3385) compared to a hospital labour ward (US$4673). Effectiveness results favoured the birth centre for this low risk cohort, calculating that only when the transfer rate from the birth centre to the hospital exceeded 62%, would the cost-effectiveness reverse.

### Different uses of cost methodology

Economic analysis is broadly characterised by two features: addressing the inputs and outputs (costs and consequences) of a given activity and identifying the critical criteria that inform the decisions around how resources are distributed or spent [35]. The basic tasks of an economic analysis are therefore to “identify, measure, value and compare the costs and consequences of the alternatives being considered” (Drummond, 2005, p.9). Distinguishing between the methods of economic or cost analysis can be difficult and sometimes a matter of semantics, as the title of published economic analyses is not always indicative of the true method of analysis. [35] Studies that examine only costs but include a comparison of alternatives are called ‘cost analyses’. When costs *and* consequences are examined simultaneously, the result is a ‘cost-effectiveness’ study.[35]

Six of the eleven appraised studies performed a cost-effectiveness analysis. [21, 23, 29, 31, 33] This was the most common methodology reported with the other method being cost analysis. [14, 19, 30, 32, 34]

#### Cost-effectiveness analyses

Three of the cost effectiveness analyses in this review were published since 2012, two of which examined the cost of providing alternative birth settings at one health service or campus [23, 31] alongside an RCT [31] and a cohort study [21, 23]. All three studies took the perspective of the health care provider. Two studies used administrative data to report costs either with a ‘Cost Per Patient’ (CPP) measure [31] based on the hospital’s activity-based costing system, or direct hospital costs derived from patient records that were then aligned with Australian-Refined Diagnostic-Related Groups (AR-DRGs). [23] Toohill (2012) also derived the costs of primary health care episodes (funded federally in Australia) through a diary that was given to the participants at the start of the trial as well as the woman’s own Pregnancy Handheld Record (PHHR).

Schroeder et al. (2012) performed a cost-effectiveness study from the perspective of the National Health Service (NHS) and calculated the incremental cost and incremental effects of births planned to be in the different birth settings and were expressed as mean cost per woman in each birth setting and mean net benefit. Unit cost estimation combined top-down/ bottom-up costing methods and resources costed were listed and augmented by an expert advisory group involving focus groups of midwives. This data was obtained through the questionnaires and datasheets associated with the larger Birthplace in England Study. Bootstrapping and sensitivity analyses were used to address uncertainty around ratios and variation in costs.

The three remaining cost-effectiveness studies were published between the years 1995 and 2000. Stone and Walker (1995) and Anderson and Anderson (1999) examined the “National Birth Centre Study”. The National Birth Centre Study (1989) studied 11, 814 low-risk women admitted for labour and delivery at 84 free-standing birth centres in the United States. They were followed though their labour, birth, transfers and for at least four weeks after birth[36] A decision analytic model was devised by Stone and Walker which allowed all logical and ‘chance’ events to be considered in an analysis. Direct costs were limited to “hotel costs” (inpatient accommodation costs) and antenatal care costs by a Certified Nurse Midwife (CNM), CNM fees for labour and postpartum care, medical costs for caesarean delivery and ambulance costs for transfers. Indirect costs (for example fixed equipment and clinician education costs) included the cost of the equipment needed to open the birth centre and the annual building lease. This data was obtained from field interviews with financial managers of the obstetric department and the birth centre provided patient charge data. Local ambulance companies also reported costs of transfers.

Anderson and Anderson (1999) took the perspective of the cost to Medicaid and reported charges by CNMs from 29 US states through a survey mailed out to every CNM known to the American College of Nurse-Midwives (ACNM). The Medicaid Program in the USA provides free or low-cost medical benefits to low-income people who have no medical insurance or have inadequate medical insurance. These charges were compared to obstetrician charges from published work [37] and the Health Insurance Association of America. No resources or accommodation charges were reported in this study and the charges were obtained at different time periods, however this was adjusted for by calculating projected cost increases due to inflation.

In Canada, an observational study by Reinhartz et al (2000) used data collected from perinatal medical records, client postnatal questionnaires and physician billing information from the Regie de l’assurance du Quebec (RAMQ), the Quebec health insurance board. The costs were considered from four perspectives in total—the Health Ministry, the regional board, the client and her family. Other costs were obtained from non-physician professional associations to value visits made by women, for example a chiropractor or dietician and unit costs for drugs were calculated on generic prices where available.

#### Cost analyses/studies

Hundley et al (1995) and Kenny et al (2105) also performed an economic analysis alongside an RCT. Kenny et al (2015) estimated costs associated with medical and midwifery staff for antenatal, intrapartum and postnatal care as well as investigations and interventions and hospitalisation costs. The financial data were collected by an experienced hospital accountant and costs relating to resource use estimated and verified with financial and services managers. Midwifery managers provided advice on the various pathways women in the service may take throughout their pregnancy. Hundley (1995) performed the cost analysis from the perspective of the hospital by costing the settings separately and classifying the costs into four main groups: staff costs; consumables; capital costs; and overheads. The main method of data collection was by questionnaire to the midwife managing the birth and the women on discharge from each facility. Medical record review also elicited information regarding consumables and interventions such as monitoring, pharmaceuticals, operative delivery and vaginal examination.

In the Netherlands Hendrix et al (2009) used the Dutch manual for costing in healthcare, which is a methodological reference for performing cost studies. This six-step reference included regarding the scope of the research, the choice of cost categories (this study measured the health sector costs and non-health care costs), resources to be included, the volume of resources, the valuation of the resources, and the calculation of the unit costs. Hendrix et al took the societal perspective and collected data identifying health care costs such as contact with health care professionals, medications, medical interventions, analgesia and hospitalisation. Non health care costs were regarded as costs to the woman and family and were collected using cost diaries, questionnaires given to the women and birth registration forms. [14]

The second study in this review from the perspective of Medicaid is by Howell et al (2014). This study estimated costs based on a previous study that used propensity score reweighting and instrumental variable analysis. Major components of the cost of care were estimated for the antenatal, intrapartum and postnatal care and these included physician costs (from Medicaid fees), midwife costs (from an algorithm that calculated a proportion of the Medicaid and Medicare physician fee) and Maternal and infant hospital costs were calculated from the National Inpatient Sample and the National Perinatal Information Centre for costs incurred in the Neonatal Intensive Care Unit (NICU). Howell et al. (2014) went on to report that the most costly component of maternity care was the maternity facility cost regardless of the model of care.

The results varied among the evaluations largely due to the differing costing methods, the data collection, the measurement and valuation of the resources identified. The authors concluded in eight of the eleven evaluations that it was less costly to give birth in a setting alternate to a standard labour ward. [19, 21, 23, 29, 31–33] Two evaluations resulted in no significant difference in the cost of the alternative setting [14, 28] and one showed an increased cost; however this evaluation included the cost of the introduction of the birth centre facility. [34]

## Limitations

This review included international and Australian studies spanning two decades. Due to the variations in timing, method of economic evaluation, currency and local economies, it was not possible to meaningfully compare the outcomes of the evaluations between the jurisdictions and the Australian setting.

## Discussion

Labour and birth consume significant resources in every country and in Australia it is the primary reason for acute hospital admission. Transferring the cost of place of birth is critical for policy, practice and management. In light of this we aimed to locate and assess the quality of economic evaluations of place of birth and the different associated costs.

Systematic reviews of economic analyses can be difficult due to the varying study designs employed. [26] There have been advances in the methodology of conducting systematic reviews of economic analyses which include the use of appraisal tools [35] and guidelines on how to conduct these reviews. [38] Considering the difficulty in synthesising the diverse results obtained from economic studies from around the world, often consisting of various economic evaluation styles, the question is asked whether they have any utility. [39] There are limitations in generalising results from economic evaluations due to variations in methodology, specificity of context and local costs, decisions and services available [40], however Anderson (2009) outlined three reasons to conduct a systematic review of economic analyses. These were firstly to justify and inform decision model development to ensure there is not already a current model in effect and secondly, to identify the most relevant study or studies potentially to translate or adapt those results. Finally to identify and understand the key economic trade-offs of a given intervention that can be multi-factorial, highlighting the contextual differences contributing to final outcomes [39]. These reasons fitted with our overall aim–to review the economic evaluations or cost analyses comparing places of birth to inform future work in this area.

It is important to note that all costs in this review were reported in local currency and at varying times, thus the value of reporting the monetary values rests in the comparisons between the birth settings rather than the generalisability of the actual costs reported. In fact, with increasing health care costs and greater competition for finite resources, it would seem negligent to avoid performing economic analyses on service delivery sectors; the question is which economic analysis is going to provide the best evidence? Is economic data a driver of reform? There is demand from women to expand birthing options [4] however there is only anecdotal evidence that cost is a large factor in the reluctance of health institutions to provide alternative birth settings for these women.

This review has presented a variety of economic analyses that have been conducted on place of birth between 1995 and 2015; however the methods tend to vary in their clarity and scope of the measurement of resource use and costs included in the calculations (see Table 1). Overcoming selection bias presents a challenge in choosing the most effective methodology of economic evaluation. Ultimately the task of an economic analysis is to provide evidence to inform decisions regarding service provision where resources are finite and alternatives need to be considered.

### Where the main costs lie

#### Home birth

There is high quality evidence that midwifery-led care in pregnancy, labour and birth is safe, with comparative or improved outcomes for women at low risk of complications. [41–44] The comparative cost of birth settings is more difficult to find. How costs are measured and estimated adds to the difficulty of drawing firm conclusions regarding where the costs lie and how to make a model of care and birth setting affordable for the health system and acceptable for the women who wish to access alternative pregnancy care. These limitations included the variety of approaches to measuring and indeed valuing resource use, data sources from which this information was obtained, and the methodologies used.

Homebirth costs included midwifery time and some consumables whereas the charges in the hospital group included accommodation for the women as well as the fixed costs of facility use and variable costs including staff time and consumables. Broadly speaking, most studies included in this review reported lower costs associated with giving birth at home or in birth centres. Given the ethical implications of performing an RCT to measure the differences in cost and outcomes of place of birth, the cohort study by Schroeder et al (2012) provided the most methodologically robust results. Prospective data collection in real time allowed the data collectors to document detailed use of resources, staff time and consumables, a method that could provide high quality data in future detailed cost analyses.

#### Birth centres

The difference in costs for women attending birth centres were associated with fewer interventions and procedures [21, 31] shorter length of stay or lower accommodation costs and facility overheads. [21, 32, 33]

Cost savings can be found by moving low-risk childbearing women away from hospital settings where there are established fixed costs to consider, as well as the availability of the convenient use of technology and interventions that are motivated by increased throughput and medical practices. Logically, home births require fewer resources and do not incur accommodation costs. [45] The intervention rates and use of technology are typically lower in birth centre settings, interestingly, more so in freestanding birth centres than alongside birth centres. [21]

#### Standard labour wards

Undeniably, standard labour wards carry the highest fixed costs and staff costs. Standard labour wards are necessary but the level of care is not required for all women. Many low risk women give birth spontaneously, in Australia around 50 percent [7], and probably don’t require the level of resources available in the labour ward setting. This means there is a high capacity to shift care outside the high level care settings without compromising outcomes for mothers and babies. [7, 41] Economies of scale become less applicable for standard labour wards and more favourable for birth centres and homebirth as the numbers increase in out-of-hospital settings [46], further supporting the evidence for providing similar services for women at low risk of complications.

## Conclusion

Of the eleven studies reviewed here, eight reported a cost saving associated with giving birth in an alternative birth setting, namely at home or in a birth centre. Two of the studies found no significant difference in the cost of providing care to women who chose to give birth in an alternate setting, however the benefits for the women and the staff were notable with higher satisfaction reported by both the women and the staff working in these settings and no increase in adverse outcomes. One study reported a cost increase in providing care in a birth centre. The differences reported in these studies highlight the differences in location, health system, methods of analysis and resources included in the costing. More research needs to be conducted on cost of alternative birth settings to support the growing demand for these services for women however the difficulty in accurately measuring these costs requires careful planning of methodology if the results are to be useful to service planners.

## Supporting Information

S1 PRISMA Checklist PRISMA Checklist-Costing Alternative Birth Settings (DOC)
